# The Necessity for Making Registration Attractive

**Published:** 1919-07-12

**Authors:** 


### THE MATRONS' AND SISTERS' DEPARTMENT.

### THE NECESSITY FOR MAKING REGISTRATION ATTRACTIVE.

WHEN war broke out State Registration in America was put to the test. It was intended at first that only registered nurses should be admitted to the privilege of nursing the sick and wounded soldiers. On examination it was discovered that the ranks of registered nurses contained but a fraction of the trained nurses qualified to render the highest service. The rest had simply stood outside the State Register. They had not suffered in pocket nor in the public estimation. Their prospects were fully as good as those of the registered nurses. They *did not see the good of it*. That was all.

Who can guarantee that the same thing will not happen in this country? It must be remembered that as matters now stand the number of nurses not qualified for admission to a State Register even under very easy conditions is immensely larger than the number of those whose qualifications are perfectly sound in every way. Do we not know the public well enough to be sure that many will rather favour than not those who make out they are "just as good," though technically disqualified? We are looking forward now to the period succeeding the years of grace. There will be a great pull on the part of second-rate agencies and on the part of small and special hospitals to get unregistered nurses good employment. Their efforts will be seconded by the general practitioners. In America many doctors have an intense dislike to registered nurses, arising out of some indistinct notion that they take too much upon themselves and are prone to criticise. All these factors make for confusion and uncertainty. To establish a costly system of registration without taking the precaution to make it so attractive that nurses will find it worth their while—indeed, indispensable—to get their names inscribed on the State Register would be to fail after full warning.

In the debate on the Registration Bill, on June 27, Major Astbury put his finger on this failure to introduce an attractive element as the weak point in the Central Committee's Bill. The analogy with barristers, medical men, solicitors, and the like is entirely misleading. Professional men when they marry do not cease to practise; women commonly do. Men bear the yoke for their whole lives, women often for a very short time, or with a long hiatus in between two earning periods. The result is to plant in the minds of women an ineradicable tendency to take their profession less seriously than men. At the time when they ought to be entering their names on the register and qualifying by examination for a successful career, a considerable proportion of nurses will always have their heads full of quite other ideas. They do not all mean to be nurses all their lives. The State Register must offer them an immediate attraction or they will find good reasons for passing it by. What attractions can and ought it to offer?

The College of Nursing is seeking to answer that question, but very wisely, in our judgment, it has rejected the appeal to lower motives of self-interest by constituting itself the almoner for the entire nursing profession and not merely for such as can show their title by registration. There are two definite and decisive ways through which the ordinary nurse and the public she serves can be made to understand the value of registration. The first is the bestowal of a title reserved for their exclusive use, easily recognisable by the public. The words "registered nurse" have no significance, and at present might mean a nurse-maid whose name had been entered at a registry office. The words "nursing sister" would carry conviction in every part of the Empire.

But when the State grants registration to nurses it must add something more, or the boon will still be incomplete. Without a protected uniform the nursing sisters will still in the eyes of the public be liable to confusion with nurse-maids or with evil-minded women disguising themselves for base purposes. There ought to be a regulation uniform and badge for all nursing sisters, which would prove their guarantee wherever they go, and the State ought to lend its protection by making it an offence for the unregistered to wear even a colourable imitation of it. If these boons be added, if nurses in the act of registration are raised to the rank of nursing sisters, with a distinctive uniform recognised by the State, then the register will become a live force worthy of the nursing profession, a source of strength to the new health services of the nation. Without such privileges the register will become merely the preserve of a certain section of nurses. In other words it will be a costly failure.

